# Fluorescence-Gated Flow Cytometry Approach for Measuring Lipid Flippase Activity in Mammalian Cells

**DOI:** 10.1007/s00232-026-00392-5

**Published:** 2026-08-21

**Authors:** Klara T. Scholtissek, Chris Van den Haute, Filip K. Pamula, Joseph A. Lyons

**Affiliations:** 1 Department of Molecular Biology and Genetics, Aarhus Universitet, Aarhus, Denmark; 2 Aligning Science Across Parkinson’s (ASAP) Collaborative Research Network, Chevy Chase, MD 20815 USA; 3 Laboratory for Neurobiology and Gene Therapy, KU Leuven, Leuven, Belgium; 4 Leuven Viral Vector Core, KU Leuven, Leuven, Belgium; 5 Interdisciplinary Nanoscience Center (iNANO), Aarhus Universitet, Aarhus, Denmark

**Keywords:** NBD-labeled lipid uptake assay, Fluorescent protein tags, Flow cytometry, Flippase interactors

## Abstract

P4‑ATPase lipid flippases establish transbilayer lipid asymmetry in eukaryotic membranes, with increasing evidence indicating a role for interacting partners in regulating flippase activity. However, assessing interactor‑dependent effects on flippase activity in mammalian cells is complicated by heterogeneous protein expression and background lipid transport. Here, we introduce an expression‑based gating strategy for in‑cell NBD‑lipid uptake assays that improves sensitivity and interpretability under transient expression conditions. Using this approach, we examined the influence of the putative interactors VAMP3 and TMEM230 on the ability of ATP11C to transport phosphatidylserine (PS). VAMP3 exhibited no detectable effect on NBD‑PS uptake, whereas TMEM230 produced a strong inhibitory phenotype. The expression‑based strategy increased resolution between ATP11C variants and substantially reduced the number of events required for reliable analysis. Together, these advances provide a framework for systematic, cell‑based investigation of regulatory interactions governing mammalian P4‑ATPase function.

**Graphical Abstract:**

**Figure:**
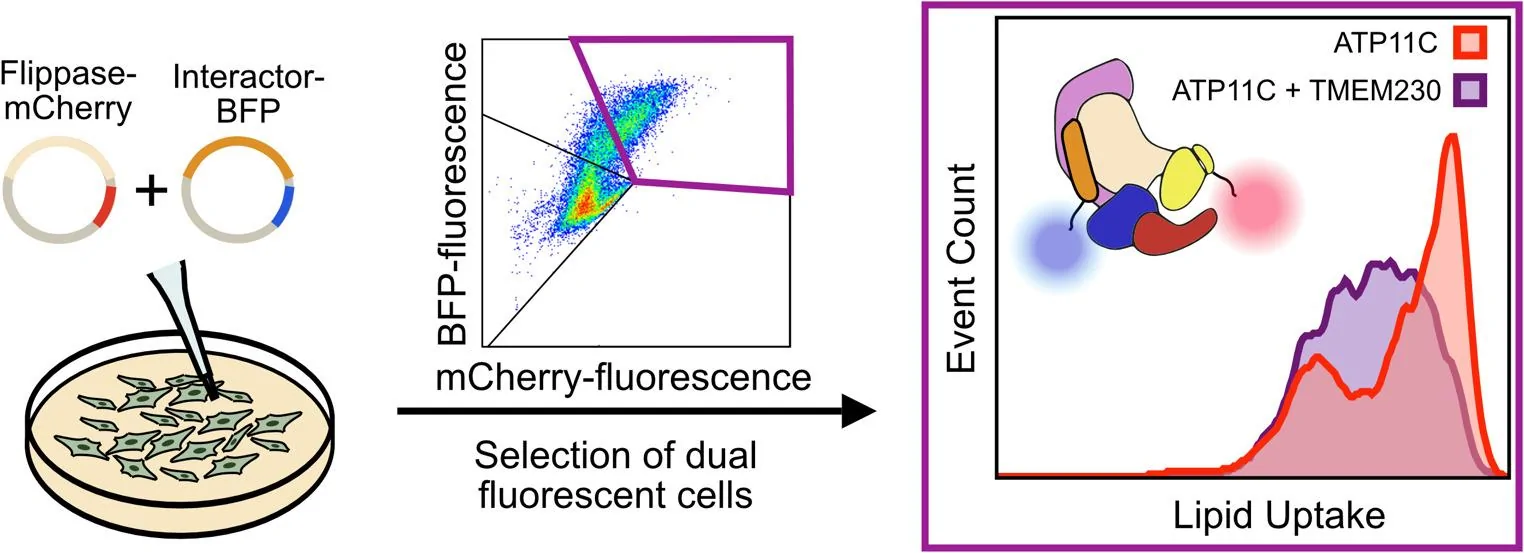

**Supplementary Information:**

The online version contains supplementary material available at 10.1007/s00232-026-00392-5.

## Introduction

A defining feature of eukaryotic lipid membranes is the asymmetric lipid distribution between bilayer leaflets, which is critical for a plethora of membrane localized functions such as membrane protein function, vesicular transport, and signal transduction (Panatala et al. 2015, Lorent et al. 2020). This asymmetric distribution of lipids is actively maintained and regulated by distinct protein families, including scramblases, floppases, and flippases. Lipid flippases (P4-ATPases) are members of the P-type ATPase superfamily and transport lipids from the exoplasmic to the cytosolic leaflet in an ATP-dependent manner (Montigny et al. 2016). Most lipid flippases are trafficked and function as heterodimers together with a β-subunit from the cell division cycle (CDC)50 family (CDC50A or CDC50B in humans) (Bryde et al. 2010, Lenoir et al. 2009, Takatsu et al. 2011). Flippase activity is highly regulated with several mechanisms reported to date (Chantalat et al. 2004, Segawa et al. 2014, Takatsu et al. 2017, Dieudonné et al. 2022, Nakano et al. 2008, Azouaoui et al. 2017). The physiological importance of P4-ATPases has become increasingly apparent, as mutations in several family members have been linked to a range of pathological conditions (Norris et al. 2024). For example, mutations in ATP11C are responsible for blood and liver disorders such as congenital hemolytic anemia (Liou et al. 2019, D’Onofrio et al. 2026, Arashiki et al. 2016).

**Fig. 1:**
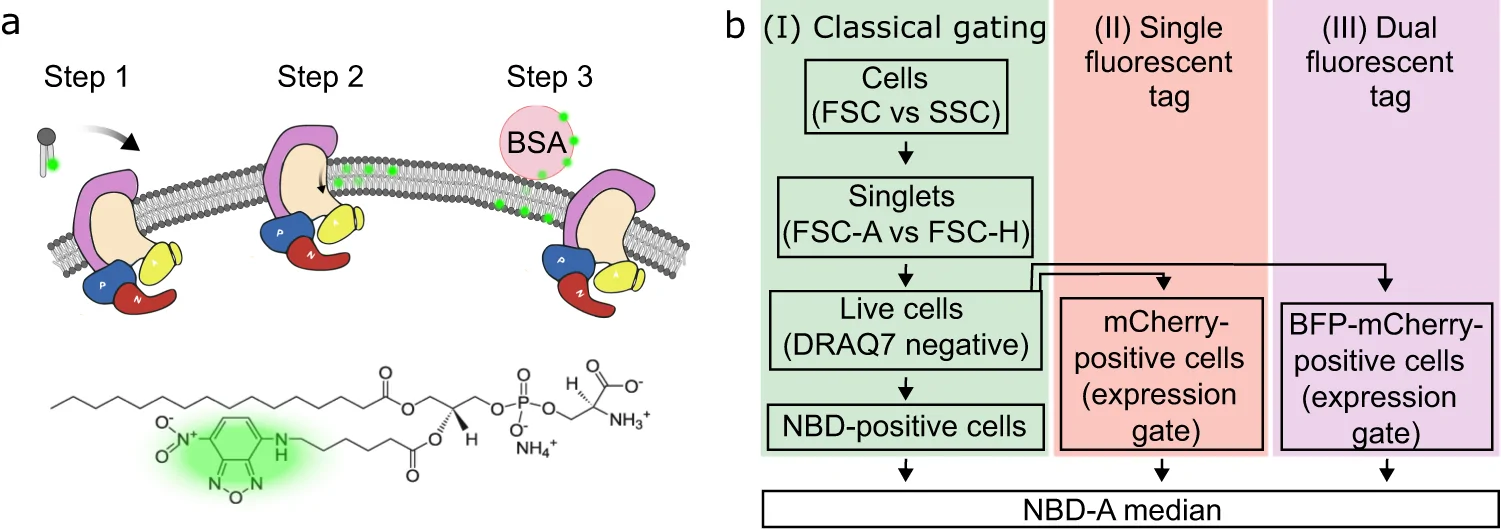
Workflow of the in-cell lipid uptake assay and comparison of gating strategies. (**a**) Schematic workflow describing the steps performed in the in-cell lipid uptake assay, including back extraction by BSA and the structure of NBD-PS with the NBD-group shadowed in green. The flippase is localized to the plasma membrane and incorporation of NBD-labeled substrate lipid is facilitated by the increased solubility of the NBD-moiety (Step 1). Flippase activity translocates the NBD-tagged substrate into the inner leaflet (Step 2) and the non-transported and unincorporated lipids are back-extracted by fatty acid free bovine serum albumin (BSA) (Step 3). (**b**) Flowchart illustrating the classical gating strategy (I, green) compared to the improved gating strategies using a single (II, red) or a dual fluorescent tag (III, purple). FSC, forward scatter; SSC, side scatter. FSC/SSC gates were used to exclude debris and select the main cell population. DRAQ7 is a membrane-impermeable DNA dye used to identify dead or membrane-compromised cells, which were excluded before quantification. For all approaches, the readout is the median NBD-A signal from the respective target gate, corresponding to NBD-uptake

Recent high-throughput proteomic analyses have identified novel protein interaction partners for P4-ATPases. The vesicle associated VAMP3 has been identified as a potential interacting partner of both ATP11C and ATP9A using endogenous tagging, live-cell confocal imaging, mass spectrometry, and data-driven analyses (Cho et al. 2022, Galli et al. 1998). In addition, TMEM230 has been proposed as a novel subunit associated with ATP8 and ATP11 flippases, supported by crosslinking approaches and native-gel mass spectrometry combined with computational modelling (Gonzalez-Lozano et al. 2025). However, the physiological and functional relevance of such interactors remain undefined. Evaluating the physiological roles of these interactors requires their study in a cellular context, where trafficking, membrane localization, and the native lipid environment are preserved. Fluorescent lipid uptake assays are widely used to measure flippase‑mediated transbilayer lipid transport in cells (Fig. 1a) (Okamoto et al. 2020, Poulsen et al. 2015, Pomorski et al. 1996). Most commonly, phospholipids labeled at the *sn*-2 acyl chain with a 7‑nitrobenz‑2‑oxa‑1,3‑diazol‑4‑yl (NBD) fluorophore are used as probes (Muranaka et al. 2024, Dieudonné et al. 2023, Herrera et al. 2022). These assays provide a direct transport readout, in contrast to indirect ATPase‑based measurements. However, current implementations have limitations for investigating interactor‑dependent effects. Stable expression systems are labor‑intensive to generate, inflexible for variant or interactor screening, and susceptible to expression level drift, whereas transient transfection systems introduce expression heterogeneity and added labor and resource costs. In addition, gating solely on NBD‑positive events can be confounded by background uptake of fluorescently labeled lipids, which hampers reliable exclusion of non‑expressing cells and complicates detection of low activity or inactive variants. Finally, these assays typically require large event counts for robust quantification, constraining practical, side‑by‑side comparisons across multiple conditions. Together, these limitations make conventional NBD‑lipid uptake assays poorly suited for systematic assessment of how interacting proteins influence flippase activity.

To address these challenges, we have developed a modified, flow‑cytometry–based NBD‑lipid uptake assay that uses fluorescently tagged flippase and candidate interactors to enable expression‑gated measurements under transient transfection conditions. This design restricts analysis to flippase-expressing cells and, with a second fluorescent tag, permits selective evaluation of cells co‑expressing a candidate interactor. These features enhance the sensitivity of the assay by eliminating non‑expressing cells from the analysis and by allowing uptake measurements to be normalized relative to flippase expression levels within the gated population. Together, they provide a flexible and scalable system suited for evaluating putative regulatory proteins whose functions remain unknown. Here, the human P4-ATPase ATP11C is used as a model to demonstrate the capabilities of the assay, both individually and when co-expressed with putative interacting proteins in a mammalian expression system.

## Results

### Fluorescent Protein Tags Improve the Selection of Positive Events

To improve the reliability of NBD‑lipid uptake measurements under transient expression, we implemented an expression‑gated flow‑cytometry approach that selectively analyzes cells expressing a fluorescently tagged flippase. Transient transfection produces heterogenous expression that complicates the interpretation of in-cell NBD-lipid uptake measurements, where classical viability- or uptake-gating strategies cannot exclude non‑expressing cells, resulting in elevated background and reduced sensitivity (Fig. 1b (I)). Here, we establish a framework in which flippase activity is measured only in cells verified to express the tagged protein (Fig. 1b (II)). For this purpose, the human lipid flippase ATP11C was fused to mCherry (emission at 610 nm), a fluorophore selected for its spectral compatibility with NBD (emission at 540 nm, Supplementary Fig. 1g) and its suitability for unambiguous detection of expressing cells. This enabled an improved gating strategy building on the classical gating (I) by incorporating an expression gate (II + III) based on either a single or a double fluorescent tag (Fig. 1b). To further improve methodological robustness and ease transient transfection requirements, we used a previously reported cell line, HeLa-CDC50A, which stably expresses CDC50A, the essential β-subunit required for the proper folding and trafficking of most human P4‑ATPase lipid flippases (Martin et al. 2020).

**Fig. 2:**
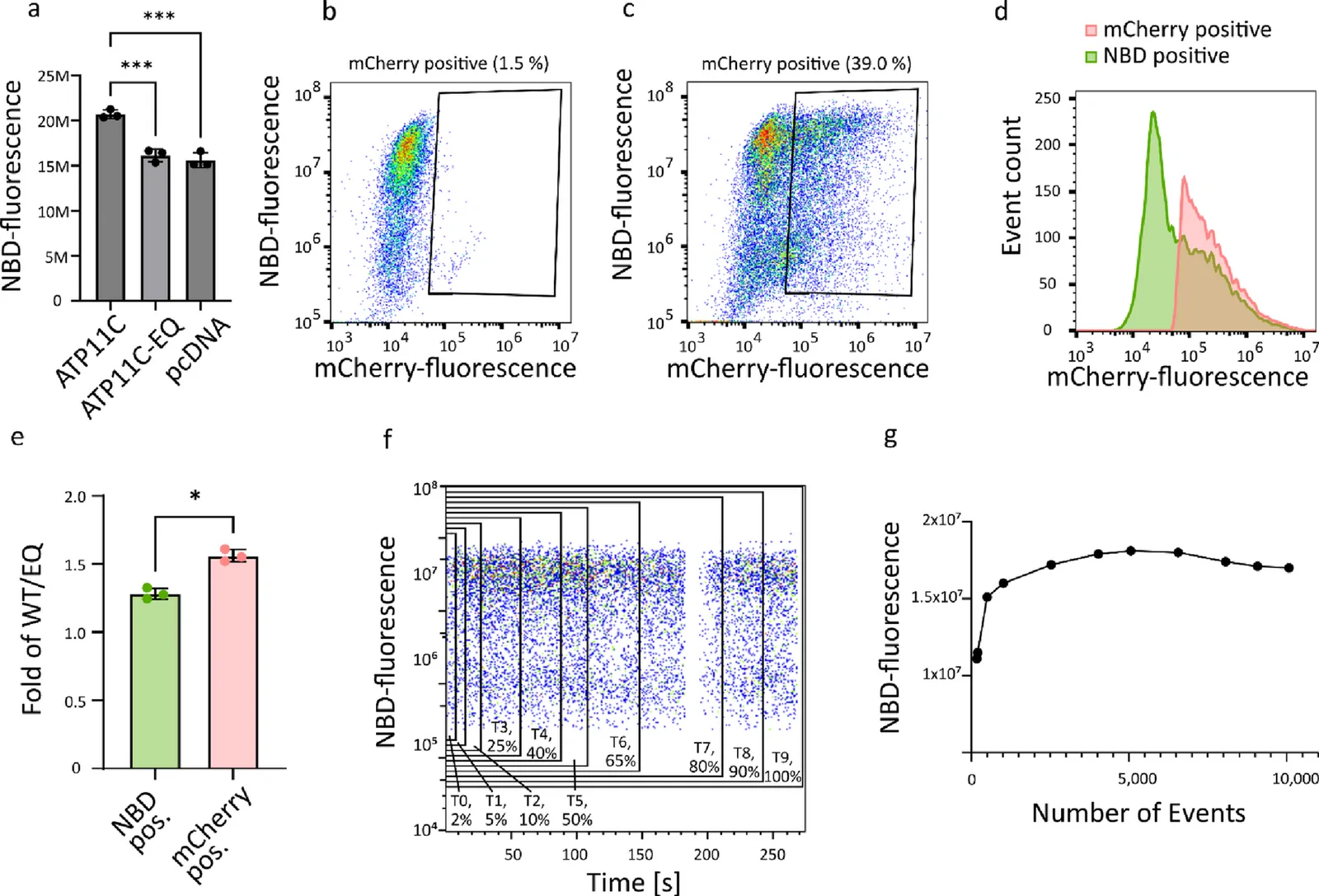
Comparison of the classical gating approach to the new fluorescent tagged approach and subsequent analysis of required events in the target gate. (**a**) Median NBD fluorescence in million (M) from DRAQ7‑negative cells gated by the classical (NBD‑positive) approach for HeLa-CDC50A cells transfected with ATP11C‑mCherry, ATP11C^E181Q^-mCherry, and pcDNA mock control (*N* = 3). Ordinary one‑way ANOVA with Dunnett’s multiple‑comparisons test (comparing to ATP11C): ***p < 0.001. (**b** and **c**) Dot plot of DRAQ7‑negative HeLa-CDC50A cells showing mCherry fluorescence (x‑axis) versus NBD fluorescence (y‑axis). The mCherry‑positive gate is indicated in dot plots from (b) pcDNA mock transfected and (**c**) ATP11C-mCherry transfected cells. (**d**) Histogram of mCherry fluorescence from HeLa‑CDC50A cells transfected with ATP11C‑mCherry. After NBD‑lipid uptake and DRAQ7 exclusion, the classical NBD‑positive gate (green, gate shown in Supplementary Fig. 1d, 13,215 events) is compared to the mCherry‑positive expression gate (red, 8,465 events). (**e**) Fold NBD‑PS uptake of ATP11C‑mCherry relative to ATP11C^E181Q^-mCherry using either the NBD‑positive gate or the mCherry‑positive expression gate as in (**d**). *p < 0.05, paired Student’s t‑test. (**f**) NBD fluorescence plotted against acquisition time for all events within the ATP11C‑mCherry expression gate, illustrating event distribution across time gates (T0–T9). The percentage of events included in each time gate is indicated. Sample acquisition happened at 180 s. (**g**) NBD-fluorescence (median) as a function of the number of events from the expression gate, demonstrating stabilization of the measurement at lower event counts

All NBD-PS uptake measurements were performed at 15 °C to minimise background endocytosis. To evaluate whether a C‑terminal fluorescent tag preserves function and to establish a gating strategy suited for transient expression, NBD‑phosphatidylserine (NBD‑PS) uptake was measured for ATP11C fused C‑terminally to mCherry (ATP11C‑mCherry) in a stable CDC50A expressing HeLa cell line (HeLa-CDC50A). Consistent with prior work on C‑terminally HA-tagged ATP11C (Takatsu et al. 2017), ATP11C‑mCherry exhibited robust NBD‑PS uptake relative to both the catalytically inactive ATP11C^E181Q^‑mCherry and the mock‑transfected control (pcDNA) (Fig. 2a), confirming that the tag does not abrogate transport.

We next compared expression‑resolved with classical gating. To distinguish transfected from non-transfected cells, a mCherry‑based expression gate (Fig. 2b and c) was used to define a discrete mCherry‑positive population comprising ~ 34–41% of all events (mean ~ 38%). Within this gate, the NBD signal (median fluorescence intensity) provided a more sensitive separation (1.6-fold) of ATP11C and ATP11C^E181Q^ than either the DRAQ7‑negative (1.4-fold) or the NBD‑positive gating (1.3-fold) strategies (Fig. 2e and Suppl. Figure 1f). Back‑gating revealed substantial contamination of the classical gated population by non‑expressing cells: 35% of events in the NBD‑positive gate and 61% of events in the DRAQ7‑negative gate were mCherry‑negative (Fig. 2d, Supplementary Fig. 1e). Together, these data indicate that expression‑based gating increases assay sensitivity and reduces misclassification arising from background uptake.

NBD-lipid uptake assays typically rely on stable cell lines or expensive transfection reagents to ensure a high number of flippase-expressing events for analysis with common practice in the field typically relying on 10,000 events. To determine the number of events required for accurate estimation of the median NBD fluorescence intensity, we performed a down sampling analysis. The NBD fluorescence intensity of individual events was plotted as a function of acquisition time (Fig. 2f) and the median NBD intensity was recalculated using progressively larger subsets of events corresponding to the defined time segments T0–T9, where T0 contained ~ 200 events and T9 represented the full dataset (approximately 10,000 events). The median NBD fluorescence intensity stabilized after 2,000–3,000 events were included (T3), yielding values indistinguishable from those obtained using the full event set (Fig. 2g). These results indicate that approximately 2,500 events are sufficient for accurate quantification, enabling reliable uptake measurements even under transient transfection conditions. All datasets presented in this study were therefore generated using a minimum of 2,500 and up to 10,000 events within the final gate, with the majority comprising 5,000–8,000 events.

### Expression‑Based Double Gating Isolates Co‑expressors for Interactor Analysis

**Fig. 3:**
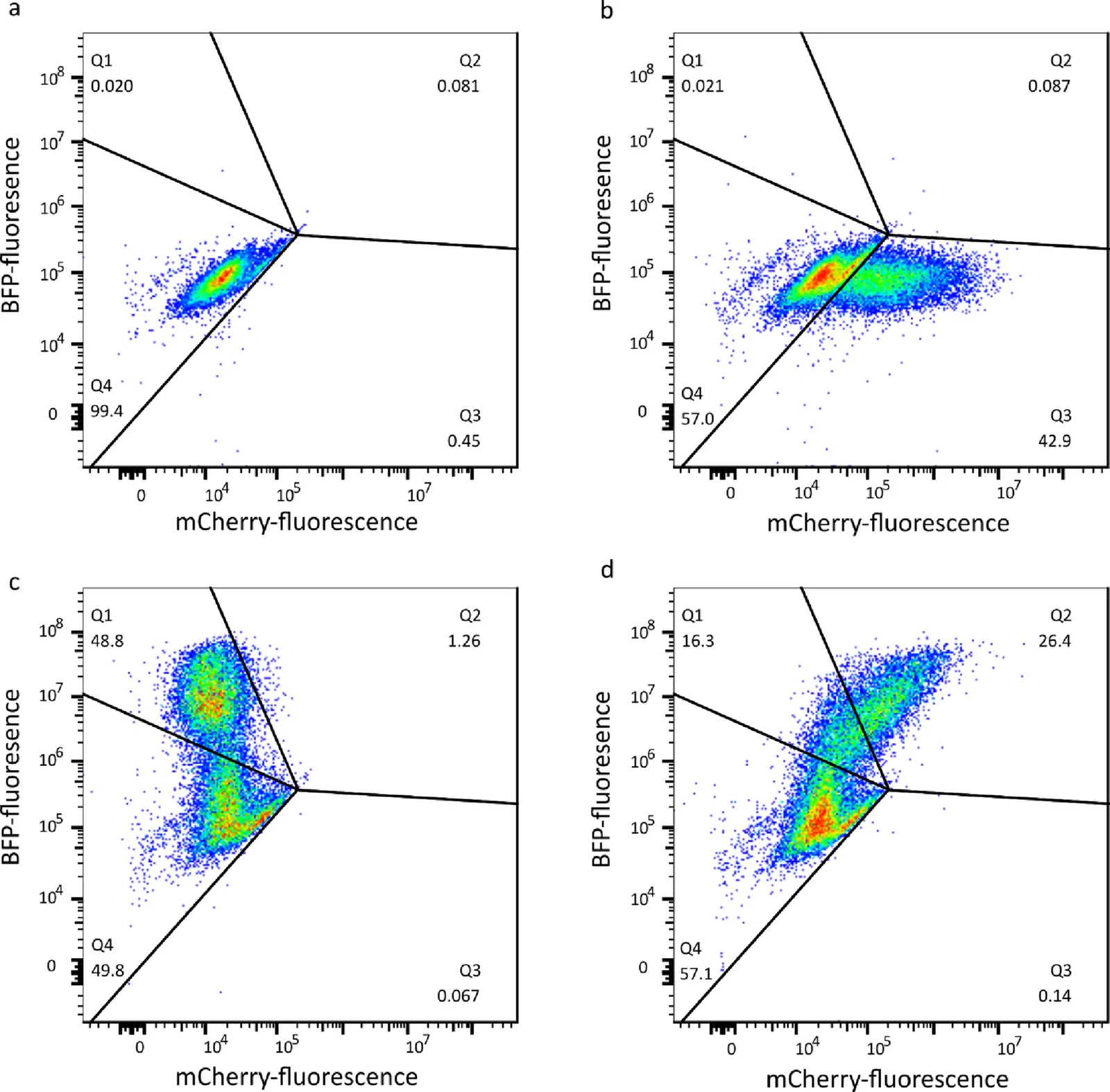
Gating strategy to select cells expressing the targets of interest. Dot plots of DRAQ7-negative events with mCherry-fluorescence (x-axis) and BFP-fluorescence (y-axis). The quadrant gate is defined to separate the non-transfected population, identified in the pcDNA mock transfection (a, Q4), from the single fluorescent populations (mCherry or BFP alone, b with Q3 or c with Q1) or the dual fluorescent population (mCherry and BFP, d with Q2). Samples shown are: (**a**) pcDNA, (**b**) ATP11C-mCherry, (**c**) BFP-TMEM230, (**d**) ATP11C-mCherry cotransfected with BFP-TMEM230. Percentage of parent population is stated below the respective quadrant

To facilitate the measurement of NBD-PS uptake in co‑expressing cells, candidate interactors were fused to BFP, chosen for its spectral compatibility with both NBD and mCherry with the aim to utilize a double‑positive (mCherry + BFP) gate (Q2) to isolate the co‑expressing sub-population (Fig. 1b (III), Fig. 3d, Supplementary Fig. 1g) (Subach et al. 2008). Expression of BFP-TMEM230 or BFP-VAMP3 was readily detected by flow cytometry (Fig. 3c, Supplementary Fig. 2a), with transfection efficiencies averaging 49% and 43%, respectively. When co-transfected with ATP11C-mCherry, we identified a discrete double-positive (mCherry + BFP) population (Q2), representing ~ 20% (ATP11C + TMEM230) and ~ 22% (ATP11C + VAMP3) of all cells (Fig. 3d and Supplementary Fig. 2b), indicating efficient co-expression. Within this double‑positive gate, we quantified the NBD-fluorescence as a direct measure of lipid uptake in cells expressing both ATP11C-mCherry and the BFP-tagged candidate interactor.

### ATP11C-Mediated NBD-PS Uptake is Inhibited by TMEM230 but Unaffected by VAMP3

**Fig. 4:**
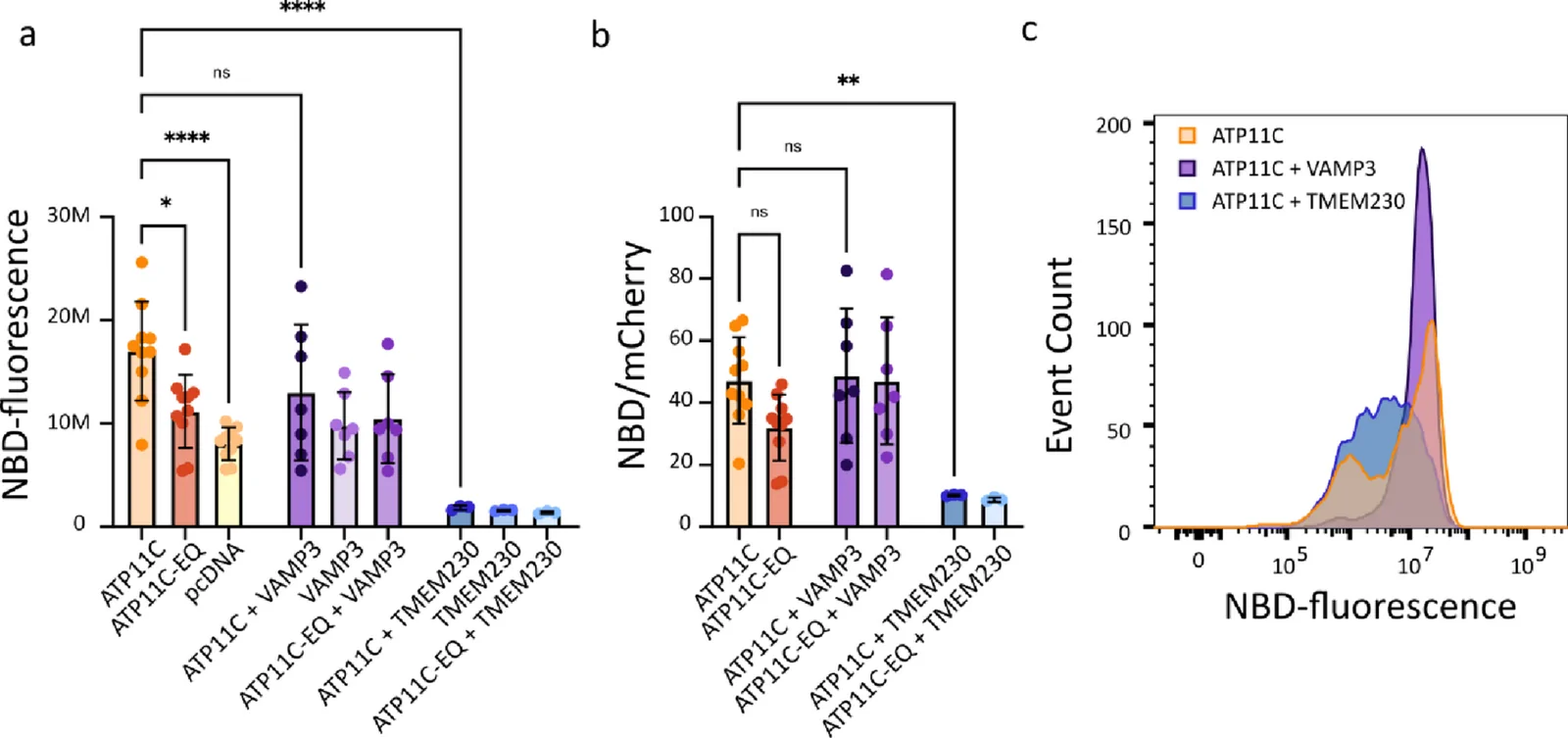
NBD-PS uptake by ATP11C co-expressed with VAMP3-BFP or BFP-TMEM230. (**a**) Median NBD-fluorescence of HeLa-CDC50A cells expressing the indicated constructs, analyzed with the appropriate quadrant gate, defined in Fig. 3. Single transfection conditions (*N* = 10) were analyzed in Q3 (ATP11C-mCherry, ATP11C^E181Q^-mCherry) or Q4 (pcDNA control). Co-expression conditions with VAMP3-BFP (*N* = 7) or BFP-TMEM230 (*N* = 3) were analyzed in Q2 (ATP11C-mCherry or ATP11C^E181Q^-mCherry with BFP-tagged protein) and the corresponding single-BFP controls in Q1 (VAMP3-BFP or BFP-TMEM230; *N* = 7 and *N* = 3, respectively). (**b**) NBD fluorescence normalized to mCherry fluorescence for the respective samples. (**c**) Representative histograms of NBD fluorescence for ATP11C-mCherry alone (orange, Q3), ATP11C‑mCherry + VAMP3‑BFP (purple, Q2), and ATP11C‑mCherry + BFP‑TMEM230 (blue, Q2) within the respective quadrant. Event counts were equalized to allow direct comparison of NBD-fluorescence distribution (3,864 events). All biological replicates consisted of technical duplicates. Statistical significance was determined using ordinary one‑way ANOVA with Dunnett’s multiple‑comparison test (comparing all conditions to ATP11C-mCherry). ns *p* > 0.05, *p < 0.05, **p < 0.01, ***p < 0.001, ****p < 0.0001

Co-expression with VAMP3 did not alter ATP11C-dependent NBD-PS uptake. The NBD fluorescence in double-positive gated cells was comparable to the ATP11C control (Fig. 4a) and normalization of NBD-PS uptake to total ATP11C‑mCherry expression supported no difference between the two conditions (Fig. 4b). Quantification of the BFP/mCherry fluorescence ratios demonstrated substantially higher BFP signal, confirming strong VAMP3 expression in the co-expressing population (Suppl. Figure 2c). VAMP3 expression alone did not affect basal NBD-PS uptake, as VAMP3-expressing cells exhibited similar NBD fluorescence compared to mock-transfected cells. Collectively, these data indicate that VAMP3 did not detectably alter endpoint ATP11C-mediated NBD-PS uptake under the expression and assay conditions tested. Because this flow-cytometry assay does not report subcellular localization, we cannot exclude that VAMP3-dependent effects require specific compartmental overlap, altered timing, or localization conditions not captured in this experiment.

In contrast to VAMP3, TMEM230 co‑expression reduced ATP11C‑mediated NBD-PS uptake. BFP-TMEM230 was efficiently expressed in CDC50A-HeLa cells both alone and with ATP11C-mCherry, at expression levels comparable to VAMP3-BFP (Fig. 3c and d compared to Supplementary Fig. 2a and b). TMEM230 co-expression resulted in a 6.7‑fold decrease in NBD-PS uptake relative to cells expressing ATP11C alone (Fig. 4a), a decrease readily apparent in the NBD-fluorescence histogram (Fig. 4c). To account for variation in ATP11C-mCherry expression arising from co-transfection, NBD-PS uptake was normalized to mCherry fluorescence (Fig. 4b). The trend in reduction in NBD-PS uptake remained significant after normalization, indicating that the lower ATP11C level does not explain the reduction in PS uptake. TMEM230 expression alone also reduced NBD‑PS uptake relative to mock‑transfected cells, indicating an effect on basal uptake mediated by endogenous lipid transport. Together, these findings identify TMEM230 as an inhibitory modulator of ATP11C‑mediated lipid transport.

## Discussion

Here, we establish a novel expression‑based gating strategy for in-cell NBD‑lipid uptake assays that improves the accuracy and interpretability of flippase-mediated lipid transport measurements. This approach enables lipid uptake measurement under heterogeneous expression conditions, allowing testing of putative flippase interactors via transient expression.

A key advantage of the assay is its improved performance in the presence of variable expression levels. The expression-based gating strategy increased assay sensitivity relative to classical uptake- or viability-based gating by excluding the negative contributions from non-expressing cells (Fig. 2d, Suppl. Figure 1e). These non-expressing cells typically have a significant background fluorescence due to endogenous lipid transport machinery which can obscure weak or modest differences in activity. This is particularly relevant for PS transporting flippases i.e., ATP11C, where NBD-PS exhibits a high basal uptake which leads to inclusion of almost all events (Suppl. Figure 1d, 67.6%). By isolating the relevant expressing population, this gating strategy produced clearer separation between active and inactive ATP11C variants, supporting improved sensitivity for low‑activity samples, negative controls, and constructs with partial loss of function. Down sampling analyses further demonstrated that reliable quantification can be achieved with markedly fewer events (2,000–3,000 events) than typically required, reducing the need for large sample sizes and making the assay compatible with transient transfection and more economical transfection conditions (Fig. 2f and g). Finally, the dual‑gating approach enabled selective analysis of co‑expressing sub-populations (Fig. 3), which is essential for interactor‑dependent modulation of flippase activity.

Using this dual-gating strategy in HeLa-CDC50A cells, we examined two proposed interactors of ATP11C, VAMP3 and TMEM230. We found that VAMP3 did not alter ATP11C-mediated or basal NBD‑PS uptake (Fig. 4), indicating no detectable functional influence under the conditions tested. VAMP3 was selected because it is a vesicle-associated SNARE linked to recycling/endosomal and plasma-membrane trafficking pathways. However, the present assay does not determine whether BFP-VAMP3 co-localizes with ATP11C-mCherry at the plasma membrane in HeLa-CDC50A cells. Therefore, the absence of a detectable VAMP3 effect should be interpreted as absence of a detectable effect on endpoint NBD-PS uptake under the conditions tested, not as evidence that VAMP3 cannot regulate ATP11C in other spatial or temporal contexts. In contrast, TMEM230 produced a robust inhibition in ATP11C-dependent NBD-PS uptake that persisted after normalization to ATP11C expression (Fig. 4). TMEM230 expression alone also reduced basal NBD-PS lipid uptake, suggesting an influence on endogenous PS transport.

Several mechanisms may account for the TMEM230‑dependent inhibition. Previous studies have linked TMEM230 as an auxiliary subunit for select lipid flippases, raising the possibility that a direct interaction could modulate ATP11C activity or affect substrate access (Gonzalez-Lozano et al. 2025). Alternatively, TMEM230 may influence the abundance of ATP11C at the plasma membrane by altering trafficking, or retention. ATP11C is known to undergo endocytosis and recycling through early and recycling endosomes under non-apoptotic conditions (Takatsu et al. 2017). Additionally, TMEM230 has been implicated in endosomal trafficking through interactions with VPS35-positive endosomes and Rab11-positive recycling endosomes (Deng et al. 2016). These observations are consistent with a role in modulating trafficking.

Our uptake data do not distinguish direct modulation of lipid transport from changes in ATP11C localization, surface abundance, trafficking, or compartmental overlap with the candidate interactor. This limitation applies to both TMEM230 and VAMP3. Confocal co-localization with plasma-membrane and endosomal markers, surface-abundance assays, and complementary in vitro activity measurements will be required to determine whether the observed TMEM230-dependent inhibition reflects direct modulation of ATP11C activity or altered trafficking/localization.

Several avenues exist for extending our new gating strategy. Plasma membrane-enriched analysis could be implemented by combining expression gating with imaging flow cytometry, where morphology‑based membrane masks allow uptake analysis to be restricted to the plasma‑membrane region and normalized to the plasma membrane‑proximal pool of ATP11C (Dominical et al. 2017). The assay can also be adapted to analyze defined sub-populations, such as cells with differing expression levels of candidate interactors or cells at specific cell‑cycle stages. Introducing brighter alternatives for mCherry fluorophore (e.g. mScarlet3) would help for even more robust separation and selection of flippase-expressing cells (Gadella et al. 2023). Incorporating titratable promoters or inducible expression systems would allow systematic modulation of flippase or interactor expression to probe dose‑dependent regulation. The increased flexibility of the assay lends itself to higher-throughput screening approaches through the evaluation of larger interactor libraries, flippase variants, or inhibitor screens. Furthermore, the assay can be easily deployed to other cell types, to examine flippase activity in a more native environment.

Despite these advantages, several methodological caveats should be considered. The assay relies on fluorescent protein fusions, and although ATP11C-mCherry retained robust activity relative to the catalytically inactive mutant, tag position and linker design may affect protein localization, trafficking, interactions, or activity. In addition, the assay reports endpoint NBD fluorescence after BSA back-extraction and assumes a negligible contribution of lipid metabolism on the measured fluorescence signal under experimental conditions. Consequently, the approach does not resolve transport kinetics or absolute internalized lipid fraction, and more quantitative questions will require modified approaches or complementary assays as described in the literature (Herrera et al. 2022). Interpretation of interactor effects is further limited by the lack of spatial resolution, as discussed for VAMP3 and TMEM230. Finally, although measurements were performed at 15 °C to reduce endocytosis, residual endocytic uptake and low-temperature effects on membrane or protein dynamics cannot be excluded. These limitations can be addressed in future studies by combining time-resolved uptake assays with co-localization microscopy, surface-abundance measurements, and endocytosis controls.

In summary, this study presents the use of expression-based gating on NBD-lipid uptake analysis to enable reliable readout of flippase activity and its modulation by putative interactors under transient transfection conditions. We show that TMEM230 has an inhibitory effect on ATP11C-mediated and basal PS uptake, whereas VAMP3 showed no detectable influence in this system. Future studies focused on ATP11C localization, surface abundance, and interaction analyses will be important for defining the underlying mechanism of TMEM230‑dependent inhibition. In general, the assay provides a flexible platform for dissecting regulatory interactions across the P4‑ATPase family and for extending studies of flippase biology in living cells.

## Materials and Methods

### Materials

Fetal bovine serum (FBS) was purchased from Biowest (SOOPF2001N). Pen Strep (P/S) (15140-122) and HBSS (#14175095) without calcium, without magnesium or phenol red were purchased from Gibco. Hieff Trans Polyethylenimine Linear MW 25 kDa (PEI) was purchased from Yeasen (40815ES03-EN). Dulbecco’s modified eagle’s medium high glucose (DMEM) (D5796), Dulbecco’s phosphate buffered saline (PBS) (D8537), Trypsin – EDTA solution (T3924) and fatty acid free bovine serum albumin (BSA) (#126609) were purchased from Sigma-Aldrich. Hygromycin B was purchased from Roche (10843555001). NBD-Phosphatidylserine (18:1 6:0) was purchased from Avanti (#810194). DRAQ7 was purchased from Invitrogen (D15106).

### Construct Design

Plasmids and primers used in this work are listed in Supporting Tables 1 and 2. All PCR reactions were carried out with Q5^®^ High-Fidelity DNA Polymerase according to the manufacturer’s protocol. Codon-optimized DNA encoding a short isoform of human TMEM230 (UniProt: Q96A57-1) was synthesized de novo and cloned into pcDNA3.1 plasmid backbone with N-terminally (N-mCherry-TMEM230) and C-terminally located mCherry (C-term-TMEM230-mCherry) construct versions by Azenta. The sequence of monomeric blue fluorescent protein (BFP) was amplified with primers Oligo1&2 and Oligo3&4 from BFP open reading frame, which was a kind gift from Orsolya Mozner. Primers Oligo5&6 and Oligo7&8 were used for N-mCherry-TMEM230 and C-TMEM230-mCherry plasmids, to remove mCherry and linearize plasmids (Linearized TMEM230). Linearized plasmids obtained this way were combined with respective amplified BFP fragments using GeneArt™ Gibson Assembly HiFi Cloning Kit according to the manufacturer’s protocol. VAMP3 sequence was amplified using Oligo9&10 from GFP VAMP3 plasmid, which was a gift from Thierry Galli (Addgene plasmid # 42310; http://n2t.net/addgene:42310; RRID: Addgene_42310). The C-BFP-TMEM230 plasmid was cut with BamHI and KasI to remove TMEM230 and create a linearized C-BFP plasmid. It was combined with the amplified VAMP3 fragment using GeneArt™ Gibson Assembly HiFi Cloning Kit according to the manufacturer’s protocol. The DNA encoding ATP11C and ATP11C E184Q were obtained from pCAG/ATP11C-HA and pCAG/ATP11C(E184Q)-HA, which were a gift from Hye-Won Shin (Addgene plasmid # 209220; http://n2t.net/addgene:209220; RRID: Addgene_209220 and Addgene plasmid # 209251; http://n2t.net/addgene:209251; RRID: Addgene_209251). Fragments encoding ATP11C were C-terminally tagged with mCherry and cloned into the pcDNA3.1 vector by Azenta.

### Cell Culture and Transfection

HeLa cells stably expressing CDC50A (HeLa-CDC50A) were cultured in DMEM supplemented with 10% FBS, 1% P/S and 160 µg/ml hygromycin at 37 ^◦^C in a humidified incubator with 5% CO_2_ and maintained in T75 flasks. For experiments, cells were seeded to reach approximately 70% confluency in 12-well plates. Adherent cells were washed once with PBS and detached using trypsin for 5 min at 37 ^◦^C. Trypsinization was stopped by the addition of DMEM. Cells were counted and diluted to a density of 90,000 cells/ml in supplemented DMEM, and 1 mL of the cell suspension was seeded into each well of a 12-well plate. Plates were gently rocked to ensure even distribution and incubated for 16–24 h at 37 ^◦^C and 5% CO_2_. Transient transfection was performed using PEI. For each well, 1 µg of plasmid DNA was mixed with 55 µL serum-free DMEM, while 5 µg PEI was diluted separately in 55 µL serum-free DMEM. For single transfections, 1 µg of plasmid DNA encoding the flippase or the empty vector (pcDNA) was used per well. For co-transfection experiments, a total of 1 µg DNA was maintained but distributed at a 4:1 ratio in favor of the flippase construct (ATP11C: VAMP3/TMEM230). In control conditions expressing only VAMP-3-BFP or BFP-TMEM230, the same DNA ratio was preserved by supplementing with pcDNA to maintain a constant total DNA amount. After incubation for 15 min at room temperature, the PEI solution was added to the DNA solution, vortexed, and incubated for an additional 15 min to allow complex formation. Cells were washed once with PBS and incubated with 300 µL serum-free DMEM before addition of the transfection mixture. Cells were incubated overnight at 37 ^◦^C and 5% CO_2_. The following day, the transfection medium was replaced with fresh DMEM supplemented with 10% FBS and 1% P/S. Expression of fluorescently labeled constructs was monitored by fluorescence microscopy.

### Lipid Uptake Assay

Transfected cells were washed once with PBS and incubated in 460 µL HBSS. Cells were equilibrated for 20 min at 15 ^◦^C prior to lipid addition. NBD-labeled NBD-PS (1 µM) prepared in HBSS and pre-equilibrated to 15 ^◦^C was added to the cells and incubated for 20 min at 15 ^◦^C. To remove non-transported and unincorporated lipids, the buffer was replaced with HBSS containing 2.5% fatty acid free BSA and 15 mM EDTA, thus halting NBD-lipid uptake. From this step onward, cells were kept on ice. Cells were detached by pipetting, filtered through a cell-strainer mesh (40–50 μm) into flow cytometry tubes, and stained with DRAQ7 to a final concentration of 3 µM to allow discrimination of dead cells.

### Flow Cytometry

Flow cytometric analyses were performed on a NovoCyte Quanteon 4025 flow cytometer (Agilent Technologies, Santa Clara, CA), equipped with four lasers (405, 488, 561, and 637 nm) and 25 fluorescence detectors. The lasers are separated in time, therefore no compensation for spectral overlap was necessary. Overview of fluorophores, lasers and filters used are presented in Supplementary Table 3. Instrument control and initial data processing were performed using NovoExpress (version 1.6.2). Samples were maintained on a cooling block during analysis, and technical duplicates were measured sequentially which were averaged. The final gate contained from 2,500 to 10,000 events.

### Data Analysis

Flow cytometry data was analyzed using FlowJo (v.10.10.1 and v11, Waters Biosciences). Cell debris was excluded based on forward scatter (FSC) and side scatter (SSC) density plots using a threshold of 150,000 and gating around the main cell population. Doublets were excluded by plotting FSC-A versus FSC-H or SSC-A versus SSC-H and selecting singlet populations. Dead cells were excluded by gating out DRAQ7-positive events. Transfected cells were identified by fluorescence of the reporter proteins. Density plots of mCherry fluorescence versus BFP fluorescence were used to distinguish non-transfected cells from cells expressing the fluorescent constructs (mCherry positive gate or respective quadrant gate). Lipid uptake was quantified as the median NBD fluorescence (NBD-A) within the gated transfected population. When required, NBD fluorescence values were normalized to mCherry fluorescence to account for differences in protein expression levels between constructs.

## Supplementary Information

Below is the link to the electronic supplementary material.

Supplementary Material 1

## Data Availability

The data, protocols, and key lab materials used and generated in this study are listed in a Key Resource Table alongside their persistent identifiers at Zenodo (10.5281/zenodo.20830163) and protocols.io (https://dx.doi.org/10.17504/protocols.io.bp2l6zo8kgqe/v1, https://dx.doi.org/10.17504/protocols.io.rm7vz44m8lx1/v1 and https://dx.doi.org/10.17504/protocols.io.bp2l6jjkkvqe/v1). No code was generated for this study; all data cleaning, preprocessing, analysis, and visualization was performed using FlowJo v.10.10.1, v11 and NovoExpress version 1.6.2.
