# Supplementary material for: Fluorescence-Gated Flow Cytometry Approach for Measuring Lipid Flippase Activity in Mammalian Cells
Source: J Membr Biol. 2026 Aug 21;259(1):32. doi: 10.1007/s00232-026-00392-5 (PMC13498495; doi:10.1007/s00232-026-00392-5)
Supplement: Supplementary file 1 [file 232_2026_392_MOESM1_ESM.docx]

**Fluorescence-Gated Flow Cytometry Approach for Measuring Lipid Flippase Activity in Mammalian Cells**

Klara T. Scholtissek^1 2^, Chris Van den Haute^2 3 4^, Filip K. Pamula*^1 2^, and Joseph A. Lyons*^1 2 5^

^1^ Department of Molecular Biology and Genetics, Aarhus Universitet, Aarhus, Denmark

^2^Aligning Science Across Parkinson’s (ASAP) Collaborative Research Network, Chevy Chase, MD, 20815

^3^ Laboratory for Neurobiology and Gene Therapy, KU Leuven, Leuven, Belgium

^4^ Leuven Viral Vector Core, KU Leuven, Leuven, Belgium

^5^ Interdisciplinary Nanoscience Center (iNANO), Aarhus Universitet, Aarhus, Denmark

email Joseph A. Lyons: [lyons@inano.au.dk](mailto:lyons@inano.au.dk)

email Filip Pamula: [fpamula@mbg.au.dk](mailto:fpamula@mbg.au.dk)

**Supplementary Information**

**Supplementary Fig. 1 Classical gating strategy compared with expression-based gating for HeLa-CDC50A cells transfected with ATP11C-mCherry.** (a) FSC-A vs SSC-A in million (M) dot plot showing all events measured and the cell gate (threshold FSC-A 150,000). (b) FSC-A vs FSC-H in million (M) dot plot showing all events from the cell gate (Cells) and the singlet gate (Singlets). (c) DRAQ7 fluorescence vs SSC-A fluorescence in million (M) of singlets with a live gate selecting DRAQ7 negative cells. (d) NBD-fluorescence of live cells vs SSC-A fluorescence in million (M) with NBD-positive gate. (e) Histogram of mCherry-fluorescence of live population (blue; 21,706 events) vs mCherry positive population, defined in Fig. 2b and c, (red; 8,465 events). f: Fold of NBD-PS uptake by HeLa-CDC50A cells transfected with ATP11C-mCherry over ATP11C-EQ-mCherry. Comparison between DRAQ7 negative population (blue) and mCherry positive population (red) with a paired student’s t test performed. Significance is indicated by asterisks, ns p>0.05, N=3. (g) Emission spectra of the four fluorophores used in the experimental set up.

**Supplementary Fig. 2 Gating strategy to select cells expressing the targets of interest.** Dot plots of DRAQ7-negative events with mCherry-fluorescence (x-axis) vs BFP-fluorescence (y-axis), using the same quadrant gating strategy as in Fig. 3. Samples shown are: a) VAMP3-BFP, b) ATP11C-mCherry + VAMP3-BFP.

**Supplementary Fig. 3 Expression of ATP11C-mCherry in comparison to VAMP3-BFP and BFP-TMEM230 within the expression gate.** Analysis was performed on DRAQ7‑negative events within the expression gate defined in Fig. 3. **a:** Median mCherry-A fluorescence intensity. **b:** Median BFP-A fluorescence intensity. **c:** Ratio of BFP over mCherry fluorescence for the indicated conditions.

**Supplementary Table 1 Overview of plasmids used and generated in this study.**

| **Plasmid name** | **Plasmid backbone** | **Insert** | **Reference** |
| --- | --- | --- | --- |
| N-mCherry-TMEM230 | pcDNA3.1 | TMEM230 N-terminally tagged with mCherry | This work |
| C-mCherry-TMEM230 | pcDNA3.1 | TMEM230 C-terminally tagged with mCherry | This work |
| N-BFP-TMEM230 | pcDNA3.1 | TMEM230 N-terminally tagged with BFP | This work |
| C-BFP-TMEM230 | pcDNA3.1 | TMEM230 C-terminally tagged with BFP | This work |
| C-BFP-VAMP3 | pcDNA3.1 | VAMP3 C-terminally tagged with BFP | This work |
| ATP11C-mCherry | pcDNA3.1 | ATP11C C-terminally tagged with mCherry | This work |
| ATP11C-EQ-mCherry | pcDNA3.1 | ATP11C E184Q mutant C-terminally tagged with mCherry | This work |
| pEGFP VAMP3 | pEGFP-C3 | VAMP3 | Ref  [PubMed 9614185](https://www.ncbi.nlm.nih.gov/pubmed/9614185) [1] |
| pCAG/ATP11C-HA | pCAG | ATP11C C-terminally HA tagged | Ref [PubMed 21914794](https://www.ncbi.nlm.nih.gov/pubmed/21914794) [2] |
| pCAG/ATP11C(E184Q)-HA | pCAG | ATP11C(E184Q) C-terminally HA tagged | Ref [PubMed 21914794](https://www.ncbi.nlm.nih.gov/pubmed/21914794) [2] |

**Supplementary Table 2 Overview of primers used in this study.**

| **Name** | **Target** | **Sequence** |
| --- | --- | --- |
| Oligo1 | BFP ORF | 5’-GAATTCAGCGAGCTGATTAAG-3’ |
| Oligo2 | BFP ORF | 5’-GCCATTAAGTTTGTGCCC-3’ |
| Oligo3 | BFP ORF | 5’-GAATTCAGCGAGCTGATTAAG-3’ |
| Oligo4 | BFP ORF | 5’- GAATTCATTAAGTTTGTGCCC-3’ |
| Oligo5 | N-mCherry-TMEM230 | 5’-GCACAAACTTAATGGCGGCGGAAGCAG-3’ |
| Oligo6 | N-mCherry-TMEM230 | 5’-CTTAATCAGCTCGCTGAATTCGCCGCTGCTG-3’ |
| Oligo7 | C-mCherry-TMEM230 | 5’-GCACAAACTTAATGAATTCGGCAGCGGTAG-3’ |
| Oligo8 | C-mCherry-TMEM230 | 5’-CTTAATCAGCTCGCTGAATTCGCCGGAGCTG-3’ |
| Oligo9 | pEGFP plasmid | 5’- CCAAGCTGGCTAGCGGATCCATGTCTACAGGTCC -3’ |
| Oligo10 | pEGFP plasmid | 5’-ATAGCACCTCCAGGGCGCCTGAAGAGACAACCC --3’ |

**Supplementary Table 3 Overview of fluorophores and the lasers used to excite them in combination with the filters used for detection on NovoCyte Quanteon Flow Cytometer.**

| **Fluorophore** | **Laser Wavelength used for excitation** | **Filter used for detection** |
| --- | --- | --- |
| Blue-fluorescent protein (BFP) | 405nm | 445/45nm |
| 7‑nitrobenz‑2‑oxa‑1,3‑diazol‑4‑yl (NBD) | 488nm | 530/30nm |
| mCherry | 561nm | 615/20nm |
| DRAQ7 | 637nm | 725/40nm |

**References**

1. Galli, T., A. Zahraoui, V.V. Vaidyanathan, et al., (1998) A novel tetanus neurotoxin-insensitive vesicle-associated membrane protein in SNARE complexes of the apical plasma membrane of epithelial cells. Mol Biol Cell. 9(6): 1437–48. <https://doi.org/10.1091/mbc.9.6.1437>
2. Takatsu, H., K. Baba, T. Shima, et al., (2011) ATP9B, a P4-ATPase (a putative aminophospholipid translocase), localizes to the trans-Golgi network in a CDC50 protein-independent manner. J Biol Chem. 286(44): 38159–38167. <https://doi.org/10.1074/jbc.M111.281006>
